# Habitat requirements of the European brown hare (*Lepus europaeus* Pallas 1778) in an intensively used agriculture region (Lower Saxony, Germany)

**DOI:** 10.1186/s12898-019-0247-7

**Published:** 2019-08-08

**Authors:** Katharina Sliwinski, Katrin Ronnenberg, Klaus Jung, Egbert Strauß, Ursula Siebert

**Affiliations:** 1 Institute for Terrestrial and Aquatic Wildlife Research, University of Veterinary Medicine Hannover, Foundation, Bischofsholer Damm 15, 30173 Hannover, Germany; 2 Institute for Animal Breeding and Genetics, University of Veterinary Medicine Hannover, Foundation, Bünteweg 17p, 30559 Hannover, Germany; 3 Present Address: Johann Heinrich von Thünen Institute, Federal Research Institute for Rural Areas, Forestry and Fisheries, Bundesallee 50, 38116 Brunswick, Germany

**Keywords:** Wildlife estimation, Citizen science, Monitoring, Hunting bags, Land use data, Small game, Habitat modelling, IACS data

## Abstract

**Background:**

The European brown hare (*Lepus europaeus*) typically resides in open habitats in agriculturally dominated landscapes in Europe. Over recent decades, a widely observed population decline occurred, which was attributed to agricultural intensification. However, with political incentives for specific crops, especially maize for energy production, the habitat went through massive changes. Thus, there is the need to identify parameters that characterize a suitable habitat for the brown hare in today’s agricultural lands.

**Results:**

We modelled European brown hare densities spatially and temporally explicit over 10 years (2005–2014) across an entire federal state. The generalized additive mixed model confirms a constant decline of the European brown hare population in Lower Saxony. Municipalities with a high proportion of grassland and precipitation totaling up to 900 mm are more favored. Woodland showed an approximately linear negative effect. The most important agricultural crop groups such as winter grains and winter oilseed rape showed overall positive effects on hare densities. However, the effect of maize was unimodal, with a positive effect of medium proportions, but a negative effect of very high proportions. The effect of sugar beet was relatively weak but negative. Brown hares were also more abundant in municipalities with a higher density of vixen with litter and municipalities with a high proportion of wildflower strips showed higher brown hare abundance.

**Conclusion:**

Lower Saxony is a diverse federal state with grassland dominated areas in the northwest, more woodland in the east, but intensive arable land in most remaining areas. The European brown hare—a species with a wide ecological potency—shows preferences to both grassland and the most typical arable crop groups such as winter grains and winter oilseed rape. The substantial increase in maize production within the time frame was likely unfavourable and may be one reason for the decline. Nonetheless, political tools such as the agri-environmental scheme “wildflower strips” were beneficial for the brown hare abundance and may be an option to reverse the decline seen over the 10 years.

**Electronic supplementary material:**

The online version of this article (10.1186/s12898-019-0247-7) contains supplementary material, which is available to authorized users.

## Background

Some wildlife species (i.e. farmland birds, small games) in agricultural landscapes are negatively influenced by intensification of agriculture [1, 2]. The change in agronomical practice is apparent in the whole of Europe with the increased mechanization, pesticide use and changes in habitats [3–5]. Especially the increase of field size, the homogenization of large fields, the removal of areas with wild vegetation and the use of pesticides have led to an incisive loss in biodiversity [6–8]. Due to the intensification in farming the consequences are increasement of uniformity and degradation heterogeneity [6].

Although, these factors are generally accepted as the main reason for the loss in biodiversity in the agricultural landscape, it is challenging to identify the importance and effect of each parameter separately [9]. A large number of studies show no monocausal reason rather than multifactorial causes that occur on a temporal and spatial scale [10].

In particular, small game species such as the European brown hare *Lepus europaeus*—a common species for an agricultural landscape is affected [3, 7]. Based on hunting bags and monitoring data a decline of the European brown hare population has been noticeable since the 1960s throughout Central and Western Europe [11–16]. However, the declining trend is not equally pronounced in every region but rather locally dependent [13, 17].

Habitat degradation was found to be the ultimate cause of hare population decline around Europe while other factors (i.e. predation, climate and disease) were proximate causes [7]. The loss of habitat quality occurred on a between-field scale (i.e. removal of non-cropped field margins) and within field scale (i.e. increasing the uniformity) [6]. In fact, European hares prefer field margins with a diverse mosaic of unimproved grassland, some crops, non-cropped areas with tall vegetation and resting places with a wide angle of side as a protection from predators. Therefore, especially field margins are an important habitat improvement for hares [18–20]. The habitat quality for hares is noticeable by their home-range size, as hares enlarge their home-range in areas with a large field size in order to include the required habitat types [1, 21, 22]. Unimproved grassland with a heterogeneous structure is strongly associated with a high number of hares [23] while it is usually lower in non-arable habitats such as grassland, forest and uplands [23–25].

Several studies concerning predation influences– especially red foxes (*Vulpes vulpes*)—imply an important focus on the population dynamics of European brown hares [10, 26, 27] while other studies could not find a significant effect [28]. Especially the impact of red foxes on leverets seems to result in different population densities [29–31]. Due to oral vaccination of red foxes against rabies in the 1990s a population regulation by disease was excluded [32]. Studies on predation pressure on hares are limited to the effect of red foxes, therefore further research on other predators, i.e. goshawk *Accipiter gentilis*, raccoon dog *Nyctereutes procyonoides*, raccoon *Procyon lotor* and carrion crow *Corvus corone* is required [33].

The European Brown hare a native inhabitant of the steppes and is negatively influenced by a high precipitation rate [14, 34] as it results in leveret mortality [35]. Mild winter conditions result in higher survival of young hares but are also accompanied by a higher mortality by facilitating the risk of disease transmission [35].

To understand the hare population dynamics, a consistent long-term and large-scale monitoring is required [36]. In the past, hunting bags were suitable measures of long-term population trends, however the data needs to be considered with caution [37]. Furthermore, in areas of declining densities they fail, as hunters limit or stop hunting for hares. Additionally, the willingness, the ability of hunters and the weather conditions influence the hunting success. Thus, the hunting renouncement affects the hunting bags disproportionately [37]. The inclusion of volunteers into ecological studies is more advantageous, as it allows a new dimension of research due to collecting data on a large scale at minimal costs [38, 39]. Recording long-term data leads to insights into population dynamics and assists management decisions [40]. Furthermore, it enhances the exploring of changes in phenology, relative abundance, survival and reproductive success of organisms across time and space [41].

In 2005, the Integrated Administration and Control System (IACS) was introduced in Lower Saxony in order to control direct payments to the farmers by member states of the common agricultural policy (CAP). The data comprise of detailed information on cultivated crops and field sizes. Legislation for the promotion of renewable energy sources in Germany is based on European regulations, in particular the Directive of 2001 on the promotion of electricity produced from renewable energy sources, which was implemented in 2003. This led to a rise of biogas plants and accordingly an increase in the cultivation of maize and other energy crops since 2004 [42]. These political decisions have a direct influence on the intensely used agricultural landscape of the federal state of Lower Saxony.

In this paper we modelled the habitat effects of the European brown hare based on wildlife survey monitoring data and IACS land use data from 2005 to 2014 for Lower Saxony. We expect the positive effect of proportion of (a) wheat, (b) grassland and negative effects of proportion of (c) increasing maize cultivation to hare densities.

## Results

**Fig. 1:**
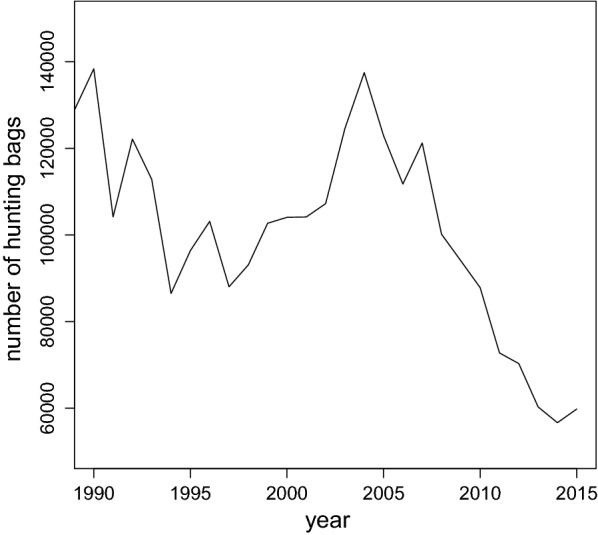
Hunting bags of the European hare from 1991 to 2015 in Lower Saxony, Germany

**Fig. 2:**
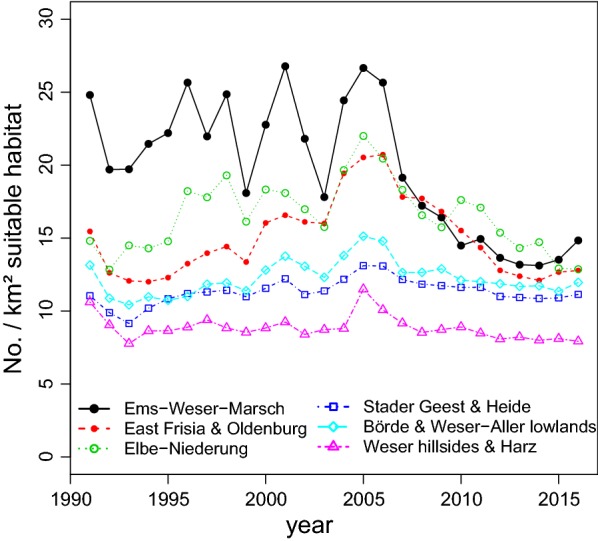
Mean number of the European hare per km^2^ open land per municipality. As part of the wildlife survey estimates are recorded through annual questionnaires of local hunters summarized for six natural regions from 1991 to 2015

**Fig. 3:**
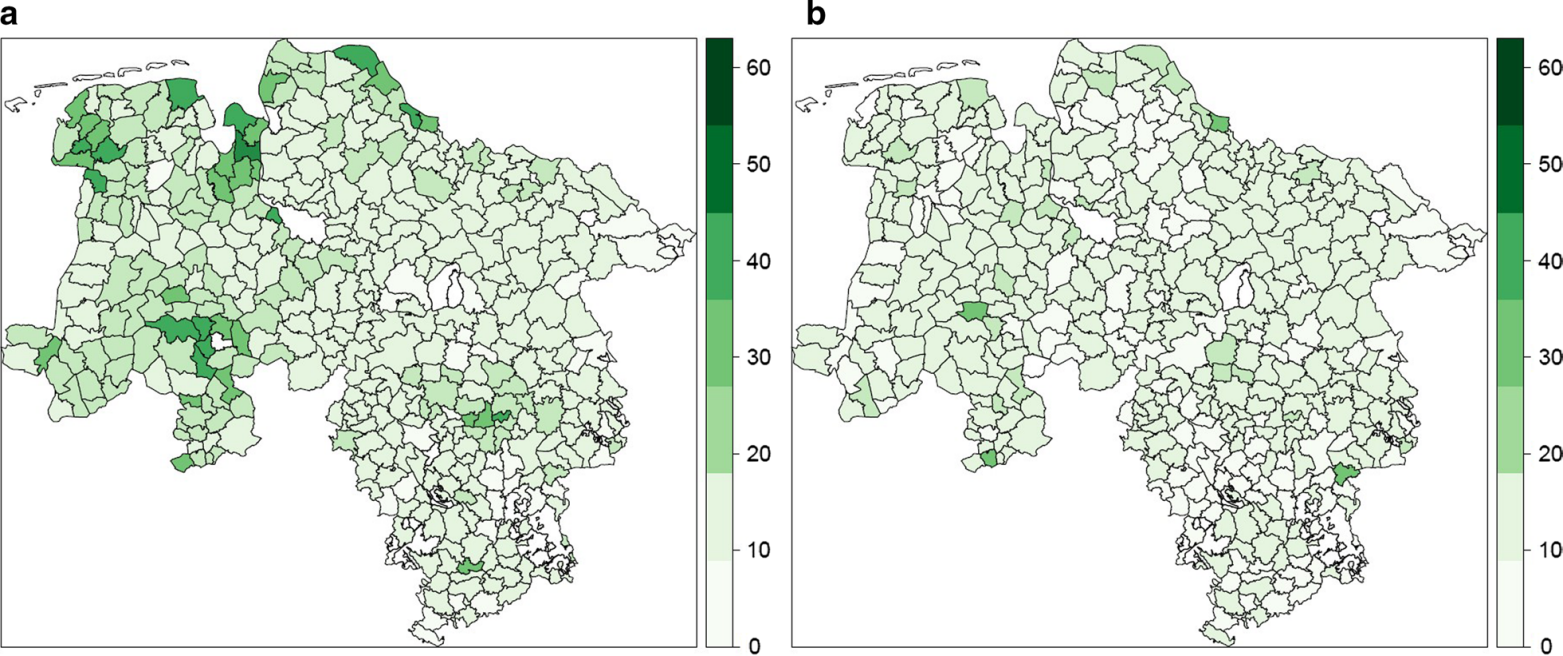
European hare density (km^2^ open land) per municipality in Lower Saxony in **a** 2005, **b** 2014

On the basis of hunting bags (from 1956 to 2015) as well as monitoring data (from 1991 to 2015), despite pronounced fluctuations, a steep decline of the European brown hare population is noticeable in Lower Saxony (Figs. 1, 2). Depending on the region in Lower Saxony, the population of the European brown hare occurs in different population densities (Fig. 3a, b). Due to regression lines in the data points for 1991–2005 and 2005–2015, respectively, separately for each of the six regions the overall trend is visible (see Additional file 1; Figure S1). Each slope of a natural region is positive for the first time period and negative for the second time period (see Additional file 1; Figure S1). Since confidence intervals don’t overlap, the change in the slopes can be regarded as significant for all six regions.

The densities range on municipality levels between 3 and 60 hare/km^2^. The highest densities occur in the intensively farmed arable areas in “Börde”, “Ems Hunte Geest” and Dümmer Geest-Niederung” as well as in the grassland areas of “Fluss- und Seemarschen” on the coastline of the North Sea. The monitoring data showed that the average population densities have increased from 11.0 to 16.9 hares/km^2^ between 1995 and 2005. Supra-regional population crashes have been noticeable since 2006, whereby, they have reached numbers of 11.3 hares/km^2^. The dramatic decline is particularly apparent in areas with originally high densities (western and northern parts) and continued in the following years, until the decline stabilized itself at a low level in recent years. Areas with traditionally low hare densities (southern and eastern parts) have mostly remained constant during the same time period. In spring 2015, the population of hares ranged—depending on region—between 8.1 and 13.5 hare/km^2^.

### Habitat modelling

**Fig. 4:**
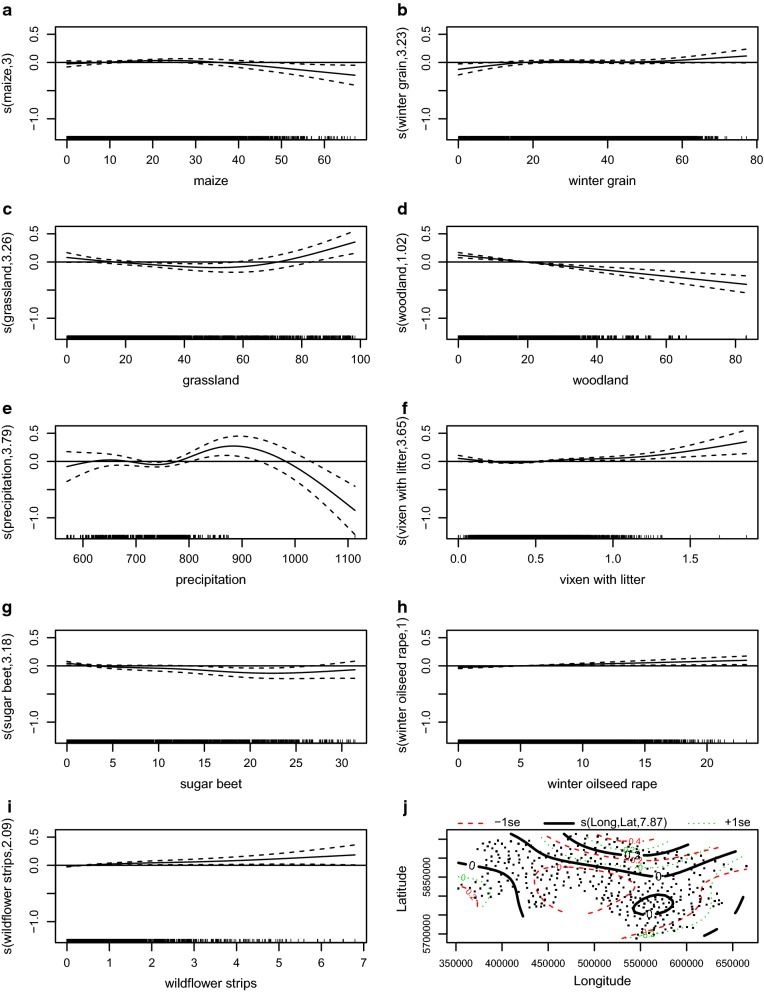
Minimum adequate habitat model of the European hare. Figure displays results of GAMM showing significant smoothers: **a** maize, **b** winter grain, **c** grassland, **d** woodland, **e** precipitation, **f** vixen with litter, **g** sugar beet, **h** winter oilseed rape, **i** wildflower strips, **j** longitude × latitude. R^2^ adjusted = 0.42

The minimum adequate model (R^2^ = 0.42) for the habitat for the European brown hare population showed a constant decline from 2005 to 2014 (Table 1). The model shows an unimodal relationship to percentage of maize per area, which is the most important smoother. With a share between approximately 10–40%, the effect of maize is moderately positive. An increase beyond that has a negative effect on the hare abundance (Fig. 4a). Fewer than circa 30% winter grain has a negative effect; higher proportions show a positive effect (Fig. 4b). According to the model, municipalities with a low proportion of grassland below roughly 70% have a negative effect for hares when compared to areas with a higher proportion (Fig. 4c). The effect of woodland shows a significant linear negative trend. The sample size of areas with more than around 50% woodland is low; therefore, the standard error is large (Fig. 4d). The precipitation rate is negatively associated up to 750 mm/year. At higher values, a positive effect was found, however a large sample size is required to draw concrete conclusions. The Harz mountains receive the highest precipitation and are an exception for Lower Saxony (Fig. 4e). The model indicates that a higher number of vixen with litter have a positive effect on hare densities (Fig. 4f). Sugar beets were continuously negatively associated with hares (Fig. 4g). At winter oilseed rape values higher than around 6%, are areas generally favourable (Fig. 4h). Wildflower strips are the second most important smoother for hare population density and show a positive effect (Fig. 4i).

**Table 1:** Summary of the GAMM-model fitted to the observed data showing the effects and their significance on hare densities

Parametric coefficients	Estimate	SE	t value	Pr(> |t|)	
(Intercept)	2.866	0.045	63.588	< 2e−16	***
factor(year)2006	− 0.026	0.013	− 1.988	4.69E−02	*
factor(year)2007	− 0.164	0.014	− 11.903	< 2e−16	***
factor(year)2008	− 0.210	0.015	− 13.772	< 2e−16	***
factor(year)2009	− 0.244	0.016	− 15.266	< 2e−16	***
factor(year)2010	− 0.273	0.017	− 16.184	< 2e−16	***
factor(year)2011	− 0.290	0.018	− 16.330	< 2e−16	***
factor(year)2012	− 0.336	0.018	− 18.449	< 2e−16	***
factor(year)2013	− 0.376	0.018	− 20.796	< 2e−16	***
factor(year)2014	− 0.377	0.018	− 20.682	< 2e−16	***

Approximate significance of smooth	edf R	ef.df	F	p-value	
s(maize)	3.568	3.568	17.780	5.22E−13	***
s(winter grain)	2.917	2.917	4.034	9.98E−03	**
s(grassland)	3.465	3.465	11.281	3.14E−08	***
s(woodland)	1.564	1.564	15.079	1.42E−05	***
s(precipitation)	3.780	3.780	5.298	2.28E−04	***
s(vixen with litter)	3.098	3.098	4.164	4.88E−03	**
s(sugar beet)	3.239	3.239	3.244	2.37E−02	*
s(winter oilseed rape)	1.000	1.000	11.454	7.20E−04	***
s(wildflower strips)	3.176	3.176	16.546	6.93E−11	***
s(Long, Lat)	8.006	8.006	5.599	4.17E−07	***

The adjusted coefficient of determination was ${\text{R}}^{ 2}_{\text{adjusted}}$ = 0.42

## Discussion

The decline of the European brown hare population can be confirmed according to hunting bags as well as monitoring data in Lower Saxony. Hence, this trend corresponds with studies on hare populations throughout Europe [7, 12, 36]. The development of the population is different in each European country as well as regionally dependent and discontinuous in Lower Saxony for the time period of 1991–2005. A significant continuous and supra-regional decline is visible since 2005. To gain further insight into wide ranging population dynamics, an advanced knowledge of habitat preferences on long term and large scale land use data is necessary as well as accurate monitoring data.

The cultivation of maize has doubled in Lower Saxony since 2004. It has increased from 300.000 to 630.000 ha in 2012 and composes a third of farmland [43]. The cultivation of energy crops is responsible for the loss of biodiversity in farmlands and especially for the decline of farmland birds [44]. The proportion of maize was the most important explanatory variable for the model. Areas in which maize is cultivates moderately affect hare abundance in a positive way and previous studies support the hypothesis of a neutral or negative effect on hare density [7, 45]. Our results confirm a negative effect starting from an amount of over 40% of maize. Hares probably benefit from maize cultivation as it serves as a structural enrichment in habitats dominated by grassland or crops as a cover opportunity, as long as they are cultivated in a modest range. The ecological impacts of bioenergy planting of *Miscanthus* crops on diet and home-range of hares was investigated in England [46]. These areas were used for resting during their inactive period. Even small proportions of energy crops had a positive effect on the home-range size and density, while huge fields lead to nearly a fivefold increase of the home-range. Further investigation on the microflora in hares on the island Pellworm (Germany) was performed due to the increase in maize production for bioenergy. A shift and decrease in diversity of intestinal flora, a coccidiosis and varying infestations was indicated, which all leads to a reduction in fitness [47]. A large cultivation of maize might force hares to fall back on maize as a food resource, which leads to a negative effect on hare abundance. A moderate change in cultivation as for example may possible result in different population reactions.

Proportion of fields planted with wildflower strips were the second most important smoother and had a linear positive effect. The positive effect of wildflower strips has been proven by previous studies on other species due to significant higher densities, i.e. insects [48] and smaller mammals [49, 50] including the European brown hare [23, 51, 52]. The positive effect on the European hare population results probably from an improved food supply and year-round cover from predation [53]. The increased proportion of permanent shelter structures improve the hare density [26]. In regards to wildflower strips, the structure is essential on leveret survival. They should offer a sufficient width to create a core area as the centre of wildflower fallows leads to the lowest prey rate of predators, while leverets on the field margins are often preyed on by predators. It is known that predator use linear structures, as the edges of wildflower strips. In conclusion, the narrower the wildflower strips, the higher the predation rate [51, 53].

Woodland is the third most important explanatory variable. The hare, as a common species for open landscapes, occurs rarely in woodland, whereby a linearly negative effect with higher proportions of woodland is expected. Nevertheless, some broad-scale studies confirm a positive association with improved grass- and woodland [23, 25]. Studies performed on local-scale showed that hares selected woodland in inactive periods as resting places [54], while other studies did not confirm these findings [55].

Winter oilseed rape has a linear positive effect on hare densities. A positive association of rape and hare density has previously been confirmed [7]. It’s not clear if winter oilseed rape is preferred as an active habitat for foraging or as a passive habitat for shelter. A histological analysis of food content in hare stomachs leads to the result that the consumption of rape was very low (0–3%) [56]. Whereas, other analysis discovered much higher amounts of this plant in the hare diet (15–39%) [57, 58]. Due to the high content of glucosinolates in winter oilseed rape during autumn an avoidance of this plant in hare diet was assumed during this time [58], while other studies indicate that winter oilseed rape is avoided in the diet in general, but hares may spend a substantial amount of their time in fields during winter [10].

The effect of grassland habitat is equivocal and contradictory. Our results show that an amount between 15 and 70% of grassland has negative effects on hare abundance, whilst a higher amount of grassland shows positive effects on hare abundance. In general, the fact established that grassland is an unsuitable habitat and the abundance of hares in these habitat types low. This was due to the reason of limited food, frequent mowing and a lack of shelter [10, 59, 60]. However, this result is not transferable to the wildlife survey data of Lower Saxony which show high densities in the grassland dominated in areas next to the North Sea [61]. The further use of the grassland also plays a role for hare occurrence. Grassland with intensive use by cattle or sheep is avoided. The amount of pasture grazing is minute in Lower Saxony, whereas the use as grass silage is more common. Consideration should be given to the aspect that different regions (i.e. differences in agricultural practice, soil conditions, climate, seed mixtures) with grassland cultivation might imply a diverse effect on hare population.

Winter grains were found to be suitable habitats for hares that was confirmed by different previous studies. Proportion of winter grains correlates positively with hare abundance in our study. Growing winter cereals are usually the most preferred diet during winter [22, 62, 63]. In a later growth stage it acts as a beneficial cover during the breeding season in spring [22]. In general, tall vegetation in spring and summer is known to have a positive effect [22]. A negative effect of winter grains during summer was assumed during harvest and this leads to a limitation as an available food resource [10]. In the time frame of our study, a judgement on an effect considering an entire year was possible, however it doesn’t include a detailed view on the seasonal vegetation status as growth height or harvested areas.

Ascending from a proportion of circa 2% of sugar beet per municipality has a continuous negative effect on the hare abundance. Controversely, other studies reveal a significant positive relation between hares and sugar beet [7, 10]. Investigation on hares diet selection assume that sugar beet fields are often used after harvest for foraging vital plant parts [62]. As shown by habitat analysis, sugar beet fields are used as food and shelter depending on its vegetation development [64]. It seems as if hares use root crops as temporary habitats because of the lack of other more adequate habitats.

In consideration of the red fox a positive effect was found with high hare densities. Hence, an effect of predation can not be substantiated. In the first place, the result seems contradictory to literature that indicates foxes as a factor for hare decline [65] or at least a limiting factor limiting for hare population [27]. A large number of studies were undertaken with regard to predator prey relations by foxes and hares. These data demonstrate the fox as a key factor for the population decline of hares [10, 27, 65, 66]. A reduction due to fox predation by hunters leads to an increase in small game species [27, 65, 67–69] nevertheless, it is not assumed to be an essential part of conservation [70]. On this broad-scale data it seems likely that habitats which are preferred by hares also present a favourable habitat for foxes. However, habitat management delivers an even more efficient alternative to predator control, as patterns of agriculture affect the predation pressure by red foxes, which have a lower predation success in heterogeneous structured fields than in homogenous structured landscapes [7, 71, 72]. This fact supports the statement that the more important criterion is the habitat structure and predation by foxes is a subordinate cause for population decline in Lower Saxony. Nevertheless, this statement is critical, as the fox density is higher in heavily wooded areas, while hares prefer arable dominated landscapes in Lower Saxony. In that respect, more research is necessary.

The precipitation rate as a minor smoother shows low hare abundance with lower values in the range of about 730 mm and higher abundance in a higher value range from 800 to 900 mm. The results stand in contrast to the common assumption that a decreasing of the hare population is connected with a higher precipitation rate [73, 74]. Another study assumed an indirect effect on soil conditions by precipitation, as good soil conditions are dry because wet soil attaches to hare feet and interferes with running [75]. Monitoring data in Switzerland reveals high hare population density despite a high precipitation rate over a long term [59]. However, farmers created compensation areas during this time. The precipitation rate as a coefficient for hare abundance seems to be embedded in a complex structure with other environmental factor like cultivars and soil condition, however further studies are required.

## Conclusion

Our study conducted the first analysis of large-scale data based on citizen science monitoring data of the European brown hare combined with land use data. A similar model has been applied on pheasant and grey partridge. The crucial factors of habitat requirements on a supra-regional scale for hares are difficult to identify and to interpret. On the one hand, our results confirm our assumptions and support previous studies, like a positive effect due to high cultivation of winter grain or a negative effect due to higher proportion of woodland. On the other hand, some of our results are equivocal based on our understanding of European hare ecology based on the current literature.

Lower Saxony is quite diverse regarding its regional scale—from coasts over lowlands to hills—and so is the land use. While the northwest is dominated by grassland, the east is dominated by woodland. However, intensive arable land is present in most areas. The European brown hare has a wide ecological potency and shows preferences for different types of land use such as grassland and the most typical arable crop groups including winter grains and winter oilseed rape. By contrast, a higher amount of maize was found to have a negative effect. Therefore, an increase in maize production over the last decade may be an important factor of numerous causes for the decline. The implementation of agri-environmental scheme “wildflower strips” has been positive throughout on the European hare, which is why it might be an effective tool to improve habitats to reverse the decline.

## Methods

### Study area

Lower Saxony is a federal state of north-western Germany with a total area of 47,620 km^2^. It reaches from the North Sea of the North German Plain to the southeast Harz mountains (up to 1000 m elevation). It is structured politically in 455 municipalities within 47 districts. The land use of the area is composed of 60.9% for agriculture, 21.6% for forest, 7.3% settlement and open space area, and 2.3% are open waters. The other parts include traffic and industrial areas.

Regarding the land use and the distribution of arable crops over Lower Saxony, huge differences are recorded. The northern and north-western parts are by far most frequently covered by grassland—in some areas with an amount of over 50%. The western and south-western areas are predominantly cultivated with wheat, in some districts closely followed by maize and potatoes. Crops followed by winter oilseed rape are the most important cultivation in the South and East of Lower Saxony, whereby the proportion of root crops is represented in the Börde. Grassland is represented very seldomly in such areas.

Lower Saxony belongs to the temperate climate zone of Central Europe with a transition area between maritime climate in Western Europe and continental climate in Eastern Europe. The average annual temperature is around 8 °C. The precipitation ranges from 500 mm/year (eastern Lower Saxony) up to 1000–1600 mm/year (in the hilly regions in south Lower Saxony) [76].

### Data

#### Wildlife survey

A long term wildlife survey WTE (Wildtiererfassung Niedersachsen) was initiated in 1991 for Lower Saxony, Germany [42, 77]. The wildlife survey, a citizen science program, is carried out by district holders and local hunters who are instructed to estimate their wildlife stock (a concrete number) yearly in spring.

Additionally, data is collected regularly about the occurrence of wildlife species and other wildlife topics. The participation rate of hunting districts ranged between 80 and 90% (6151–8300) for the years 1991–2014, whereby over 90% of the huntable area of Lower Saxony was recorded (approximately 43,000 km^2^) [42, 78, 79]. The average size of a hunting ground is approximately 500 ha (min 75 ha, max 4877 ha). The hunting district holders declare the current areas of hunting ground, wooded land and open land.

In total, the hunting area of Lower Saxony is divided into about 9000 hunting districts.

These estimations, including the European hare and the red fox are calculated on “*n* hares (or foxes) per 100 ha huntable area” and aggregated on municipality level. Outlier values (s > 5) are deleted (Outlier-test, WINSTAT). The estimations of the European Brown hare population were evaluated in 1995/96 by spotlight counts in 31 districts, and in 2004–2006 by thermographic counts in 53 districts—choosing the study areas randomly [80]. The spotlight counts were performed under standardized method [81] from March until the end of April with a spotlight, which achieves an effective illuminous range of 150 m. The mean error ratio was 1.6. The thermographic counts were conducted form 2004–2006 in 53 randomly chosen hunting grounds during spring [82]. The applied method was related to the spotlight count of Pegel but instead of a spotlight a thermography system was used. The result was an underestimation of 45% or rather an estimation by hunters of only 55% of the actual hare stock. The Mean error quotient was 1.8, which replace the previous correction factor of 1.6 retrospective for the past data as well as the future data. Based on the evaluation, estimations of district holders who do not perform a spotlight count on their ground are adjusted with a factor of 1.8 since. In addition, we used estimations of vixen with litter per km^2^ of huntable area, which are also performed by hunting district holders. Due to the availability of land use data from 2005 to 2014, we used the same time series of wildlife survey data for modelling.

The estimations and counts from all hunting districts are aggregated to municipality level in order to intersect the wildlife survey data with IACS data. Unincorporated land as well as islands in the North Sea were excluded (in total 35 municipalities).

#### IACS

The Integrated Administration and Control System (IACS) of the European Union was developed for the administration of the European agricultural direct payments. It was agreed on in 1992 as a reform of CAP and introduced to Lower Saxony in 2005. Within this regulation, data of land use concerning arable fields are aggregated of all farmers that received subsidies, which constituted 90% of all agricultural land for Lower Saxony (LEA Portal, website). IACS data were provided by the SLA (“Servicezentrum, Landentwicklung und Agrarförderung” in Lower Saxony).

The data includes land use information of individual field identification, field size, crop type and the municipality it was situated in. Due to data protection, land-use information is aggregated to greater municipalities in order to protect personalized data. The allocation of area per municipality was conducted by the SLA. For further analysis, data was summed to percentage of area agricultural land per municipality. For administrative purposes IACS data are grouped into 164 different crops. In order to receive meaningful statistics, it was summarized into ecologically useful groups (see Additional file 2; Table S1) for performing the habitat modelling.

Geographic data concerning the main landscape features as woodland, water area and grassland were provided from the LSA (Landesamt für Statistik Niedersachsen) (http://www1.nls.niedersachsen.de/statistik). Data was available for the years 2005, and 2009–2014. In order to allow an analysis over a continuous course of time, the geographic data for the missing years 2006–2008 were replaced with the values from 2005. These landscape features only changed slightly over the 10 years [42].

In order to include precipitation and temperature, the necessary data was downloaded from WorldClim global climate dataset [83].

### Statistical analyses and habitat modelling

The data preparation as well as the analyses were conducted in R (V3.1.2, [84]. Generalized additive mixed models (GAMM) were conducted using the R package ‘mgcv’ [85, 86]. The Bayesian approach for GAMM-models was used to determine significance of model parameters and thin plate regression splines where used to calculate the smoothing terms of the models [87]. Model selection on fixed effects was accomplished by AIC comparisons using maximum likelihood estimations (see Additional file 1; Table S1). As response variable, the numbers of hares/km^2^ was used. As fixed effects, the following parameters of cultivation were selected on percentage amount of each municipality: maize, winter grain, summer grain, grassland, forest, sugar beet, winter oilseed rape, wild fields and flower strips. Additionally, vixen with litter/km^2^, precipitation and temperature where chosen as parameters. Year was used as factor. In order to account for repeated measurements, municipality was included as random effect.

## Additional files

**Additional file 1.** Process of model selection for the Habitat (GAMM), regression lines for two time periods (1991–2005, 2005–2015) for mean number of the European hare per km^2^ open land per municipality, Diagnostics of the GAMM: residual distribution, Variance Inflation factor of each parameter of our GAMM.

**Additional file 2.** List of crops eligible to payments schemes between 2005 and 2014.

## Data Availability

Raw data cannot be made available since that may contradict privacy protection of hunting district holders and farmers. For scientific purpose access might be provided directly by the Ministry for nutrition, Agriculture and customer protection of Lower Saxony.

## Abbreviations

IACS: integrated administration and control system
WTE: wildlife survey lower saxony (Wildtiererfassung Niedersachsen)
EEG: renewable energy directive (Erneuerbare-Energien-Gesetz)
LEA-Portal: internet portal for rural development and agricultural subsidies (Landentwicklung und Agrarförderung)
SLA: service center for rural development and agricultural subsidies (Servicezentrum Landentwicklung und Agrarförderung)
GAMM: generalized additive mixed model
CAP: common agricultural policy
