# Supplementary material for: Habitat requirements of the European brown hare (Lepus europaeus Pallas 1778) in an intensively used agriculture region (Lower Saxony, Germany)
Source: BMC Ecol. 2019 Aug 8;19:31. doi: 10.1186/s12898-019-0247-7 (PMC6686498; doi:10.1186/s12898-019-0247-7)
Supplement: Supplementary file 1 — Additional file 1. Process of model selection for the Habitat (GAMM), regression lines for two time periods (1991–2005, 2005–2015) for mean number of the European hare per km2 open land per municipality, Diagnostics of the GAMM: residual distribution, Variance Inflation factor of each parameter of our GAMM. [file 12898_2019_247_MOESM1_ESM.pdf]

Process of model selection for Habitat (GAMM) model

Method: Starting from the full model, shown in the formula as r code, all smooth terms were removed and AIC values were compared. The model with the lowest AIC-value was taken as new model to remove smooth terms until the removal of terms could not reduce AIC values anymore. The final model is marked in fat font. Latitude*longitude and year were not removed for reasons of spatial and temporal autocorrelation.

Tab. S1: Model select selection for the European hare habitat model

formula	Model	df	AIC	BIC	logLik
gamm(log1p(number of hares) ~ s(maize)+s(winter grains)+s(grassland)+s(forest)+ s(precipitation) +s(vixen with litter)+ s(sugar beet)+ s(winter oilseed rape)+s(wildflower strips)+s(summer grains) +s(temperature)+s(Long,Lat)+, correlation=corAR1 (form=~year|municipality), random=list(municipality=~1) , method="ML")					
full model	1	41	-1026.5584	-769.0805	554.2792
- forest	2	39	-916.4949	-669.301	497.2475
- maize	3	39	-974.0844	-729.1664	526.0422
- winter grains	4	39	-1025.2239	-780.3058	551.6119
- grassland	5	39	-1000.3925	-755.4745	539.1963
- precipitation	6	39	-1027.6727	-782.7547	552.8364
- vixen with litter	7	39	-1022.1233	-777.2052	550.0616
- sugar beet	8	39	-1023.6363	-778.7182	550.8181
- winter oilseed rape	9	39	-1020.8624	-775.9444	549.4312
- wildflower strips	10	39	-995.8577	-750.9396	536.9288
- summer grains	11	39	-1029.5668	-784.6488	553.7834
- temperature	12	39	-1040.6315	-795.7134	559.3157
gamm(log1p(number of hares) ~ s(maize)+s(winter grains)+s(grassland)+s(forest)+ s(precipitation) +s(vixen with litter)+ s(sugar beet)+ s(winter oilseed rape)+s(wildflower strips)+s(summer grains) +s(Long,Lat)+, correlation=corAR1 (form=~year|municipality), random=list(municipality=~1) , method="ML")					
- forest	13	37	-925.1171	-690.5998	499.5585
- maize	14	37	-986.2211	-753.8629	530.1105
- winter grains	15	37	-1039.4119	-807.0537	556.7059
- grassland	16	37	-1010.8897	-778.5316	542.4449
- precipitation	17	37	-1024.0971	-791.739	549.0486
- vixen with litter	18	37	-1036.1036	-803.7454	555.0518
- sugar beet	19	37	-1039.2584	-806.9002	556.6292
- winter oilseed rape	20	37	-1033.5947	-801.2365	553.7973
- wildflower strips	21	37	-999.3369	-766.9787	536.6684
**- summer grains**	**22**	**37**	**-1044.0533**	**-811.6951**	**559.0266**

formula	Model	df	AIC	BIC	logLik
gamm(log1p(number of hares) ~ s(maize)+s(winter grains)+s(grassland)+s(forest) +s(precipitation) +s(vixen with litter)+ s(sugar beet)+ s(winter oilseed rape)+s(wildflower strips)+s(Long,Lat)+, correlation=corAR1 (form=~year|municipality), random=list(municipality=~1) , method="ML")					
- forest	23	35	-928.5343	-706.6936	499.2672
- maize	24	35	-989.3512	-769.553	529.6756
- winter grains	25	35	-1042.3503	-822.552	556.1752
- grassland	26	35	-1014.6323	-794.834	542.3162
- precipitation	27	35	-1027.5488	-807.7505	548.7744
- vixen with litter	28	35	-1039.477	-819.6788	554.7385
- sugar beet	29	35	-1042.1859	-822.3877	556.093
- winter oilseed rape	30	35	-1037.3304	-817.5322	553.6652
- wildflower strips	31	35	-1003.3856	-783.5873	536.6928

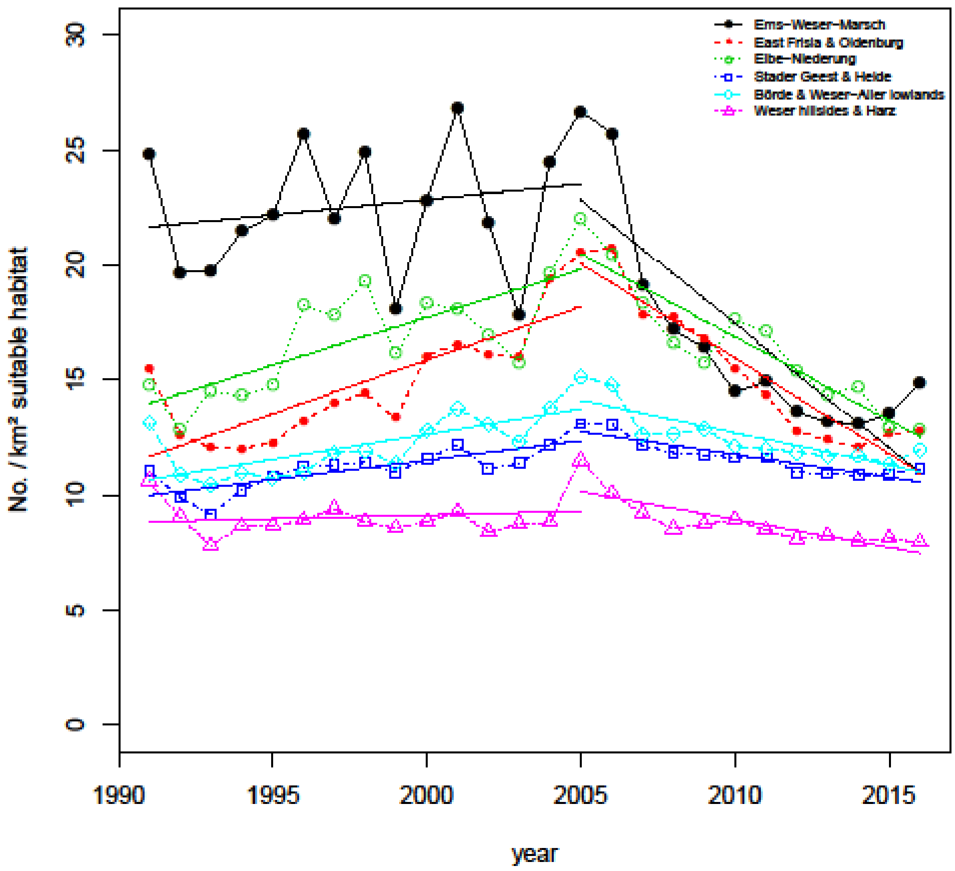

Fig. S1 Mean number of the European hare per km2 open land per municipality. As part of the wildlife survey estimates are recorded through annual questionnaires of local hunters summarized for six natural regions from 1991-2015. Including regression lines for two time periods (1991-2005, 2005-2015).

Tab. S2: Slopes of the regression lines for two time periods (1991-2005, 2005-2015) and their 95% confidence intervals, separately for each natural region.

	Regression slope 1991-2005	Slope 95% CI	Regression slope 2005-2015	Slope 95% CI
EmsWeserMarsch	0.1333	[0.0436, 0.2229]	-1.0821	[-1.2107, -0.9535]
OstfrieslOldbg&Osnab.Raum	0.4652	[0.4168, 0.5137]	-0.8309	[-0.8809, -0.7809]
ElbeNiederung	0.417	[0.3663, 0.4676]	-0.721	[-0.7731, -0.6689]
StaderGeest&Heide	0.1697	[0.1512, 0.1883]	-0.2001	[-0.2163, -0.1840]
BoerdeWeserAllerFlachland	0.2193	[0.1891, 0.2495]	-0.2803	[-0.3128, -0.2479]
WeserberglandHarz	0.0334	[0.0053, 0.0615]	-0.2417	[-0.2702, -0.2132]

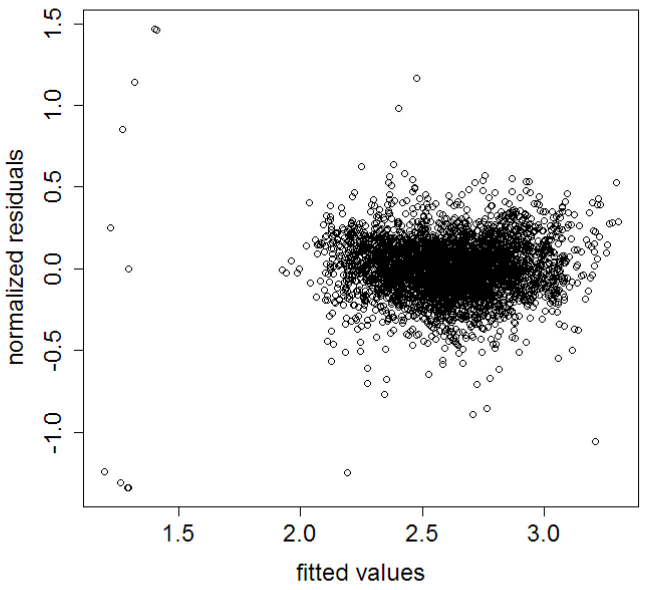

Fig. S2: Diagnostics of the GAMM: residual distribution

Tab. S3: Variance Inflation factor of each parameter of our GAMM

Parameter	Variance Inflation Factor
maize	4.67
winter grain	6.24
grassland	6.45
woodland	2.18
Precipitation	4.00
vixen with litter	1.98
winter oilseed rape	4.26
sugar beet	4.76
wildflower strips	1.52
Long	8.47
Lat	6.67
Year	1.51
