# Supplementary material for: Habitat requirements of the European brown hare (Lepus europaeus Pallas 1778) in an intensively used agriculture region (Lower Saxony, Germany)
Source: BMC Ecol. 2019 Aug 8;19:31. doi: 10.1186/s12898-019-0247-7 (PMC6686498; doi:10.1186/s12898-019-0247-7)
Supplement: Supplementary file 2 — Additional file 2. List of crops eligible to payments schemes between 2005 and 2014. [file 12898_2019_247_MOESM2_ESM.pdf]

Table S1: List of crops eligible to payments schemes between 2005 and 2014.

Cereals

Code	Nomenclature	Groups for gamm
113	Durum wheat	summer grain
114	Spelt	winter grain
115	Common winter wheat (excluding durum wheat)	winter grain
116	Common spring wheat (excluding durum wheat)	summer grain
121	Winter rye	winter grain
122	Spring rye	summer grain
125	Winter maslin	winter grain
131	Winter barley	winter grain
132	Spring barley	summer grain
142	Winter oats	winter grain
143	Spring oats	summer grain
145	Spring maslin	summer grain
155	Triticale	winter grain
157	Spring triticale	summer grain
171	Grain maize	maize
172	Corn cob mix	maize
174	Sweet corn	maize
175	Companion planting of silage maize and sunflowers	maize
176	Maize with huntable- or flower strips, which are set aside	maize
177	Maize with huntable- or flower strips that are farmed with arable crops or harvested	maize
182	Buckwheat	summer grain
190	All (other) cereals	

Protein crop

Code	Nomenclature	Groups for gamm
210	Peas for the production of grain	
220	Beans for the production of grain	
230	Sweet lupines for the production of grain	
240	Peas / beans for the production of grain	
250	Mixtures of peas / cereals	
290	all (other) pulses for the production of grain	

Oilseed crops

Code	Nomenclature	Groups for gamm
311	Winter rape for the production of grain	winter oilseed rape
312	Spring rape for the production of grain	
315	Winter turnip rape for the production of grain	
316	Spring turnip rape for the production of grain	
320	Sunflowers for the production of grain	
330	Soy beans for the production of grain	

341	Linseed for the production of grain	
342	Fibre flax	
390	All (other) oilseeds	

Plants harvested green/fodder

Code	Nomenclature	Groups for gamm
411	Forage maize (as main fodder)	maize
412	Fodder root crops (excluding mangel-wurzel, rutabaga and potatoes)	
413	Mangel-wurzel	
414	rutabaga	
421	Clover	
422	Clover grass mix	
423	Lucerne	
424	Grass as arable silage for stock feed	
426	Canary seed (*Phalaris arundinacea*)	
427	Other cereals as whole crop silage	
428	Rotational grassland	
429	All other fodder crops from arable land	

Permanent grassland

Code	Nomenclature	Groups for gamm
441	Reseeding of permanent grassland as substitute for permitted tillage of permanent grassland	grassland
451	Meadows	grassland
452	Hay meadows	grassland
453	Pastures and alps	grassland
454	Wood pastures	grassland
462	grazed sandy heaths	grassland
463	grazed boggy heaths	grassland
464	Grazed nutrient-poor grassland	grassland
465	Grazed montane grassland	grassland
466	Cut nutrient-poor grassland	grassland
467	Cut montane meadows	grassland
480	Orchard with grassland farming	grassland

Set aside

Code	Nomenclature	Groups for gamm
511	Set aside without renewable resources	
516	Set aside with annual renewable resources	
517	Set aside with perennial renewable resources	
545	Set aside according to FELEG GAL	
555	Set aside for 20 years (arable land)	
556	Afforestation according to afforestation premium after 1993	
558	Set aside for 10 years (arable land)	

563	Afforestation areas on arable land to activate payment entitlements	
564	Afforestation areas on arable land (VO(EG) Nr. 1257/99 or VO(EG) Nr. 1698/2005 as set aside VO(EG) Nr.782/2003 arable land	
565	Other set aside sites	
566	Legumes on set aside	
567	Afforestation areas on arable land to activate payment entitlements	
568	Afforestation areas on arable land without activation of payment entitlements	
569	10 year set aside VO(EG) Nr. 1257/99 site without activation of payment entitlements as set aside	
572	20 year set aside with accompanying measures as set aside without activation of payment entitlements as set aside	
580	Set aside with growth of renewable resources with declaration for own biogas plant	
583	Areas that are not arable but eligible according to Art. 34 (2b (i)) according to VO(EG) Nr. 73/2009 eligible sites	
590	Set aside with growth of renewable resources with growth and supply agreement for external biogas plant	
591	Arable land, temporarily set aside	
592	Permanent grassland, temporarily set aside	

Root crops

**Code**	**Nomenclature**	**Groups for gamm**
611	Early potatoes	
612	Other potatoes / medium and late season potatoes	
613	Industrial potatoes, starch potatoes	
614	Fodder potatoes	
615	Seed potatoes	
619	Other potatoes not for starch production	
620	Sugar beet	sugar beet
640	potatoes for starch production; contractors for Südstärke	
641	potatoes for starch production; contractors for Emslandstärke	
642	potatoes for starch production; contractors for Avebe/D	
643	potatoes for starch production; contractors for Avebe/NL	

644	potatoes for starch production; contractors for Agrana	
690	All other root crops (excluding fodder rootcrops)	

Vegetables and other industrial crops

Code	Nomenclature	Groups for gamm
710	Field grown vegetables	
711	Field grown fresh peas	
712	Field grown cauliflowers	
713	Field grown tomatoes	
715	Asparagus	
722	Flowers and non-woody ornamental plants	
723	Strawberries (field grown)	
731	Vegetables and mushrooms under glass or high accessible cover	
732	Flowers and non-woody ornamental plants under glass or high accessible cover	
733	Mushroom beds and vegetal sites in buildings other than greenhouses	
750	Hops	
761	Tobacco of group I FLUE CURED (e. g. Virgin D and hybrids …)	
762	Tobacco of group II LIGHT AIR CURED (e. g. "Badischer Burley" and hybrids …)	
763	Tobacco of group III DARK AIR CURED (e. g. "Badischer Geudertheimer" and hybrids …)	
770	Medicinal, fragrant and spice plants	
771	Herbs	
777	Artichoke	
790	All other industrial crops (excluding permanent	
791	Horticulture seeds (ornamental plants)	
792	Horticulture seeds (fruit and vegetable)	
793	Hemp	

Perennial and permanent crops

Code	Nomenclature	Groups for gamm
811	Drupes and pomes	
812	Orchards without meadow utilisation	
815	Pomes e. g. apples, pears	
816	Drupes e. g. cherries, plums	
817	Soft fruit e. g. currants, gooseberries, raspberries	
818	Common sea-buckthorn	
819	Other orchards e. g. elderberry	
824	Hazelnuts	
825	Walnuts	
830	Nurseries, excluding for soft fruit	
831	Soft fruit for propagation (in nurseries)	
845	Common osier	

846	Nurseries of ornamental trees e.g. Christmas	
847	Fast-growing wood not short rotation coppice	
848	Short rotation coppice	
850	Vineyards	
890	Other permanent crops	
891	Poplars	
892	Rhubarb	
896	Chinese silver grass (Miscanthus sinensis)	
897	Plants for energy production if no other code is is available e.g. *Agropyron elongatum*	

Other areas

Code	Nomenclature	Groups for gamm
910	Other utilised agricultural areas (e.g. browsing areas for wildlife)	wildflower strips
912	Propagation of grass seeds	
915	Flower strips and field borders (NAU – A5)	wildflower strips
918	Perennial flower strips (NAU – A6)	wildflower strips
920	home and market gardens	
923	grassland without agricultural use	grassland
924	Biotopes without agricultural use	
925	Biotopes with agricultural use	
926	trench allotment cooperative wetland	
930	Utilized water bodies and ponds	
940	all other water bodies not utilized	
941	Green manure as main crop cultivation	
953	Afforestation areas on arable land VO(EG) Nr. 1257/99	
955	Afforestation areas on arable land VO(EG) Nr. 1 257/99 or VO(EG) Nr. 1698/2005 without activation of payment entitlements	
960	Dams and dikes	
965	Uncultivated peatlands	
966	Uncultivated heathlands	
990	All other areas	
991	Farm building and track area	
992	Mining land, barrens, interfile land, ---, ecological succession areas – permanently set aside	
994	Unhitched pits, straw, fodder and dung deposits	
995	Forestry area	
996	Unhitched pits, straw, fodder and dung deposits	
997	Flower gardens, parks, recreation areas, golf	
998	forests for soil conservation	
999	Due to hardship temporarily not utilized areas	
